# Editorial: Opportunities and challenges in drug repurposing

**DOI:** 10.3389/fphar.2025.1709217

**Published:** 2025-10-08

**Authors:** Ihosvany Camps, Stephan R. Künzel, Mario Schubert, Ramendra K. Singh

**Affiliations:** 1 Institute of Exact Sciences, Federal University of Alfenas, Alfenas, Brazil; 2 Institute for Transfusion Medicine, DRK Blutspendedienst Nord-Ost gGmbH, Dresden, Germany; 3 Department for Experimental Transfusion Medicine, Faculty of Medicine Carl Gustav Carus, Dresden University of Technology, Dresden, Germany; 4 Institute of Pharmacology and Toxicology, Technical University Dresden, Dresden, Germany; 5 Allahabad University Allahabad (Prayagraj), Allahabad, India

**Keywords:** drug repurposing, drug repositioning, challenges, opportunities, artificial intelligence, bioinformatics

## Introduction

Drug repurposing, defined as the identification of new therapeutic uses for existing medications, has emerged as a transformative strategy in pharmaceutical development, offering a compelling alternative to traditional *de novo* drug discovery. While conventional drug development requires USD two to three billion and 10–17 years to bring a new medication to market, with only an approximately 11% approval rate from Phase I trials (Sun et al., 2022), drug repurposing can reduce these timelines to 3–12 years at an average cost of about USD 300 million. Repurposing clinically approved drugs helps to circumvent challenges related to toxicity and poor pharmacokinetic properties, which are major causes of clinical failure for new drug candidates. This Research Topic explores the multifaceted landscape of drug repurposing through six exemplary studies that collectively illustrate both the remarkable opportunities and persistent challenges in this rapidly evolving field (Pinzi et al., 2024).

### Mechanistic innovation in cancer treatment

A substantial contribution to this Research Topic comes from Aliabadi et al., who provide a comprehensive review of mebendazole repositioning for cancer therapy. Their analysis demonstrates how drug repurposing can address multiple mechanisms of cancer drug resistance through a single compound originally developed as an antihelminthic. The authors meticulously document mebendazole’s anticancer activity across diverse tumor types, highlighting its ability to disrupt microtubules, inhibit angiogenesis, regulate autophagy, and modulate critical signaling pathways, including the ERK and Hedgehog pathways. Notably, their work demonstrates mebendazole’s superior safety compared to conventional anticancer agents, while maintaining efficacy, a hallmark advantage of repurposing (Aliabadi et al.; Maida, 2024).

Yang et al. further advance our understanding of precision repurposing in their investigation of canagliflozin for endometrial cancer. Their study exemplifies the sophisticated mechanistic approaches now possible in drug repurposing, demonstrating how the SGLT2 (sodium/glucose cotransporter 2) inhibitor canagliflozin can overcome progestin resistance by targeting the RAR-β (retinoic acid receptor-β)/CRABP2 (cellular retinoic acid-binding protein 2) signaling pathway in endometrial cancer cells lacking thyroid hormone receptor-β. This work represents a paradigm shift from empirical to mechanism-driven repurposing strategies, utilizing computational modeling, transcriptomics, and proteomics to identify novel therapeutic applications (Yang et al.).

### Addressing rare disease challenges

Chen et al. contribute crucial insights to the repurposing of traditional Chinese medicine formulations, specifically Jiawei Suanzaoren decoction for perimenopausal insomnia. Their integrated approach, combining clinical observation with network pharmacology analysis, demonstrates how computational methods can elucidate complex therapeutic mechanisms in traditional remedies. This study is particularly significant as it addresses the growing interest in validating ethnopharmacological treatments through modern scientific approaches (Chen et al.).

The work by Somorai et al. presents a compelling case study of nicardipine repurposing for Pitt-Hopkins syndrome, a rare neurodevelopmental disorder. This bench-to-bedside approach exemplifies the potential of drug repurposing for rare diseases, where limited patient populations make traditional drug development economically nonviable. Their success in achieving measurable developmental improvements in a young patient underscores how drug repurposing can provide hope for families facing conditions without approved therapies (Somorai et al.).

## Innovative delivery systems and PARP inhibition

Molnár et al. explore the repurposing of the PARP (poly ADP-ribose polymerase) inhibitor talazoparib for psoriasis, revealing unexpected therapeutic benefits beyond the compound’s original oncological indication. Their findings, that talazoparib promotes terminal differentiation of epidermal keratinocytes while also exhibiting pro-inflammatory effects, highlight the complex and sometimes paradoxical outcomes that can emerge from drug repurposing efforts. This work emphasizes the need for a comprehensive mechanistic understanding when repositioning drugs across therapeutic areas (Molnár et al.).

The study by Alrouji et al. on HDAC8 (histone deacetylase 8) inhibitors demonstrates another dimension of repurposing research, in which virtual screening identifies existing drugs with previously unknown activities against specific molecular targets. Their comprehensive analysis of radotinib and sertindole as HDAC8 inhibitors illustrates how computational approaches can systematically identify repurposing opportunities based on target-drug interaction profiles (Alrouji et al.).

### Computational advances and market dynamics

The research presented in this Topic occurs against a backdrop of rapid computational advancement in drug repurposing. Recent developments in artificial intelligence and machine learning have significantly improved success rates by accurately predicting drug mechanisms and identifying off-target effects. The availability of extensive biomedical datasets, including genomic profiles, disease registries, and real-world evidence, provides unprecedented opportunities for cross-indication mapping and drug effectiveness modeling (Cousins, Nayar, and Altman, 2024; Maida, 2024).

The global drug repurposing market, valued at USD 35.3 billion in 2024 and projected to reach USD 51.8 billion by 2032, reflects the growing confidence in this strategy. Regulatory agencies have responded by establishing streamlined approval pathways, such as the FDA’s 505(b) (2) pathway, which enables developers to leverage existing safety and efficacy data while relying particularly on clinical evidence for new indications (Wood, Laura, 2025; Sperry and Ingber, 2024; Maximize Market Research, 2017; Vermeulen et al., 2025).

## Persistent challenges and future directions

Despite remarkable progress, significant challenges remain. The studies in this Topic collectively highlight several persistent barriers: inadequate resources for systematic repurposing efforts, intellectual property complexities that limit commercial incentives, and the fundamental challenge that pharmacological inhibitors often fail to replicate genetic perturbations of the same targets. Additionally, while computational approaches have advanced substantially, the translation from algorithmic predictions to clinical success remains limited, with many promising computational hits failing to demonstrate efficacy in clinical settings (Krishnamurthy et al., 2022; Talevi and Bellera, 2020) (Alrouji et al.).

The work presented here also reveals important methodological limitations. Drug repurposing often relies on serendipitous observations or limited mechanistic insight, which may obscure optimal dosing strategies or patient selection criteria. Furthermore, the assumption that drugs failing in their primary indication will succeed in secondary applications may not always hold, as highlighted by recent systematic analyses showing that repurposed compounds may have comparable or even higher failure rates in later development stages (Talevi and Bellera, 2020).

## Conclusions and future outlook

The studies compiled in this Research Topic demonstrate that drug repurposing has matured from opportunistic redeployment to systematic, mechanism-driven therapeutic discovery. Integrating computational approaches with traditional pharmacological and clinical methods offers unprecedented opportunities to address unmet medical needs efficiently and cost-effectively. However, realizing the full potential of drug repurposing requires continued investment in computational infrastructure, collaborative frameworks between academia and industry, and regulatory innovations that balance innovation incentives with public health benefits.

As we advance into an era of precision medicine and personalized therapeutics, drug repurposing stands poised to play an increasingly central role in pharmaceutical development. The success stories presented in this Topic—from mebendazole’s anticancer potential to nicardipine’s neurotherapeutic applications—provide compelling evidence that existing drugs harbor untapped therapeutic potential waiting to be systematically discovered and clinically validated. Future progress will depend on developing more sophisticated computational methods, establishing robust validation frameworks, and creating sustainable business models that support continued innovation in this vital area of pharmaceutical research.
