# Correction: Editorial: Opportunities and challenges in drug repurposing

**DOI:** 10.3389/fphar.2025.1735051

**Published:** 2025-11-11

**Authors:** Ihosvany Camps, Stephan R. Künzel, Mario Schubert, Ramendra K. Singh

**Affiliations:** 1 Institute of Exact Sciences, Federal University of Alfenas, Alfenas, Brazil; 2 Institute for Transfusion Medicine, DRK Blutspendedienst Nord-Ost gGmbH, Dresden, Germany; 3 Department for Experimental Transfusion Medicine, Faculty of Medicine Carl Gustav Carus, Dresden University of Technology, Dresden, Germany; 4 Institute of Pharmacology and Toxicology, Technical University Dresden, Dresden, Germany; 5 Allahabad University Allahabad (Prayagraj), Allahabad, India

**Keywords:** drug repurposing, drug repositioning, challenges, opportunities, artificial intelligence, bioinformatics

The accession date was omitted for reference “**Maida, 2024**”. It should be “Maida, J. (2024). Drug repurposing market to grow by USD 8.57 billion (2024–2028) as cancer treatment applications expand, AI-driven market transformation report–technavio. Cleveland, OH, United States: PR Newswire Website. Available online at: https://www.prnewswire.com/news-releases/drug-repurposing-market-togrow-by-usd-8-57-billion-2024-2028-as-cancer-treatment-applications-expand-aidriven-market-transformation-report—technavio-302292477.html. (Accessed September 17, 2025)”.

The accession date was omitted for reference “**Sperry and Ingber, 2024**”. It should be “Sperry, M., and Ingber, D. (2024). Drug repurposing strategies, challenges and successes. Website Technol. Drug Discov. Available online at: https://www.technologynetworks.com/drug-discovery/articles/drug-repurposing-strategieschallenges-and-successes-384263. (Accessed September 17, 2025)”.

The reference for “**Maximize Market Research, 2017**” was erroneously written as “The Global Business Consultancy Firm (2017). Maximize MARKET RESEARCH. Pune, Maharashtra, India. Maximize Market Research Pvt Ltd. Available online at: https://www.maximizemarketresearch.com/ (Accessed date on November 28, 2017)”. It should be “Maximize Market Research. (2017). Drug Repurposing Market: Strategic Approaches, Technological Advancements, Market Dynamics, and Future Growth Outlook (2025–2032). Available online at: https://www.maximizemarketresearch.com/market-report/drug-repurposing-market/273130/ (Accessed September 17, 2025)”.

The original article has been updated.

